# Paper-Based Fluidic Sensing Platforms for β-Adrenergic Agonist Residue Point-of-Care Testing

**DOI:** 10.3390/bios12070518

**Published:** 2022-07-12

**Authors:** Hongzhi Luo, Shan Liu, Lina Shi, Zhu Li, Qianwen Bai, Xiaoxin Du, Lijun Wang, He Zha, Chenzhong Li

**Affiliations:** 1 Department of Laboratory Medicine, The Third Affiliated Hospital of Zunyi Medical University (The First People’s Hospital of Zunyi), Zunyi 563002, China; hongzhiluo666@163.com; 2 Sichuan Provincial Key Laboratory for Human Disease Gene Study, Department of Medical Genetics, Department of Laboratory Medicine, Sichuan Academy of Medical Sciences & Sichuan Provincial People’s Hospital, University of Electronic Science and Technology, Chengdu 610072, China; shanliusyy@uestc.edu.cn; 3 School of Medicine, University of Electronic Science and Technology of China, Chengdu 610054, China; yishilina@163.com; 4 College of Medical Technology, Chengdu University of Traditional Chinese Medicine, Chengdu 610075, China; lizhu@stu.cdutcm.edu.cn; 5 Sichuan Jinxin Women & Children Hospital, Chengdu 610066, China; lisaqianwenbai@163.com; 6 Office of Scientific Research & Development, University of Electronic Science and Technology, Chengdu 610054, China; duxx89@uestc.edu.cn; 7 Department of Ophthalmology, The Third People’s Hospital of Chengdu, The Affiliated Hospital of Southwest Jiaotong University, Chengdu 610031, China; 8 Department of Biochemistry and Molecular Biology, School of Medicine, Tulane University, New Orleans, LA 70112, USA

**Keywords:** β-adrenergic agonists, paper-based device, lateral flow immunoassay, point-of-care testing, biosensors, microfluidics

## Abstract

The illegal use of β-adrenergic agonists during livestock growth poses a threat to public health; the long-term intake of this medication can cause serious physiological side effects and even death. Therefore, rapid detection methods for β-adrenergic agonist residues on-site are required. Traditional detection methods such as liquid chromatography have limitations in terms of expensive instruments and complex operations. In contrast, paper methods are low cost, ubiquitous, and portable, which has led to them becoming the preferred detection method in recent years. Various paper-based fluidic devices have been developed to detect β-adrenergic agonist residues, including lateral flow immunoassays (LFAs) and microfluidic paper-based analytical devices (μPADs). In this review, the application of LFAs for the detection of β-agonists is summarized comprehensively, focusing on the latest advances in novel labeling and detection strategies. The use of μPADs as an analytical platform has attracted interest over the past decade due to their unique advantages and application for detecting β-adrenergic agonists, which are introduced here. Vertical flow immunoassays are also discussed for their shorter assay time and stronger multiplexing capabilities compared with LFAs. Furthermore, the development direction and prospects for the commercialization of paper-based devices are considered, shedding light on the development of point-of-care testing devices for β-adrenergic agonist residue detection.

## 1. Introduction

Food safety and food control are of increasing concern for food markets around the world. Animal-derived food provides a substantial protein source for humans and constitutes a notable proportion of many peoples’ diets. To maintain animal husbandry practices and help to develop the industry, veterinary drugs are widely used for the prevention and treatment of diseases, as well as for the promotion of growth [1]. Unfortunately, the uncontrolled use of veterinary drugs, especially synthesized β-adrenergic agonists known as “lean meat powder”, has caused many public health problems [2] and is now an issue of concern around the world.

The synthesized β-adrenergic agonist family (“lean meat powder”) includes clenbuterol (CLE), ractopamine (RAC), salbutamol (SAL), terbutaline, and clorprenaline, and they have been widely used to treat lung disease and asthma in humans. Furthermore, members of this family can also be used to improve fat decomposition and conversion and are therefore used as feed additives to accelerate growth and increase muscle in animals [3,4]. However, long-term or high-dose intake of residual β-adrenergic agonists through meat products can cause serious and harmful physiological side effects such as myalgia, dizziness, tachycardia, nervousness, and even death [5]. β-adrenergic agonists can accumulate in animal tissues as they do not readily decompose [6]. Due to this, β-adrenergic agonists are currently prohibited as feed additives in China and most European and American countries; however, several β-adrenergic agonists continue to be illegally added for economic benefits in many countries and regions [7,8]. For example, there was a clenbuterol (CLE) event in China in mid-March 2011 which sparked serious debate about food safety in relation to β-adrenergic agonists [9]. On 19 March 2021, the Ministry of Agriculture and Rural Affairs of the People’s Republic of China issued the “Notice on Launching a Special Rectification Action” for “lean meat powder” to severely crack down on its illegal use.

Due to the potential risks of β-adrenergic agonist residues on human health, and considering the need to monitor their illegal use, sensitive wide-ranged screening and convenient assays are required to allow detection on-site. In recent years, various analytical methods have been developed for the detection of β-adrenergic agonists, including liquid chromatography (LC) [10,11], gas chromatography-mass spectrometry (GC-MS) [12], LC-tandem mass spectrometry (LC-MS/MS) [13], GC-MS/MS [14], and capillary electrophoresis [10]. However, these methods are costly, laborious, and time-consuming, which hinders their use as on-site assays. In contrast, enzyme-linked immunosorbent assay (ELISA) is a sensitive and relatively simple procedure, yet it has a high false positive rate and is difficult to use on-site.

**Figure 1:**
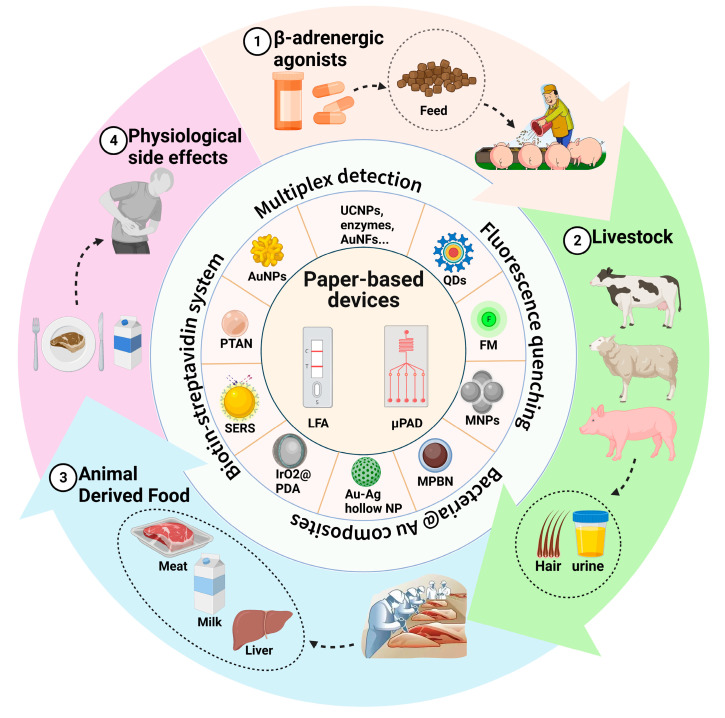
Illegal addition of β-adrenergic agonists to livestock feed poses a huge threat to public health. Various paper-based fluidic devices have been developed to detect β-adrenergic agonist residues in animal-derived food, hair, and urine from livestock, including lateral flow immunoassays (LFAs) and microfluidic paper-based analytical devices (μPADs). During the past decades, researchers have improved the detection performance of devices by developing novel labeling and detection strategies.

Paper has become a simple and reliable platform for analytical instruments, and one of the most well-known applications is the lateral flow immunoassay (LFA). Due to its unique advantages, including rapidity, portability, and simplicity, LFAs have been widely used as tests for clinical diagnosis [15,16], infectious diseases [17], and the detection of chemical contaminants [18]. LFAs combine immune-specific recognition and sensitive nano-signal characteristics, creating an excellent tool for use in point-of-care tests (POCT) for food safety. Microfluidic paper-based analytical devices (μPADs) are the newest generation of “lab-on-a-chip” devices. Since the first μPAD was released in 2007 [19], they have experienced rapid development. Using paper as a substrate for microfluidic devices has the advantages of low cost, wide availability, and simple fabrication; more importantly, externally powered equipment need not manipulate the fluid and conduct biochemical reactions. Although μPADs are still in the preliminary development stage for the detection of β-adrenergic agonists, satisfactory results have been achieved (Figure 1). Vertical flow immunoassays (VFAs) offer an improvement on the performance of LFAs due to their obvious advantages in comparison, such as shorter detection time and stronger multiplexing capability [20] and, consequently, they have the potential to be used for the high-throughput and ultra-sensitive on-site detection of “lean meat powder.”

Compared to other biological detection assays, the paper substrate, which is the main material for paper-based devices, is easy to acquire, transport, and mass manufacture. However, there are relatively few reviews on the use of paper-based devices to detect β-adrenergic agonists in animal-derived foods. In this review, we have critically evaluated the following points: (i) recent developments in LFAs for the detection of β-adrenergic agonists, including the use of new labels and signal amplification strategies; (ii) the design strategy of μPADs and their application for the on-site high-throughput detection of β-adrenergic agonists; and (iii) the future development trends for the commercialization of paper-based devices.

## 2. LFA Formats and Principles

**Figure 2:**
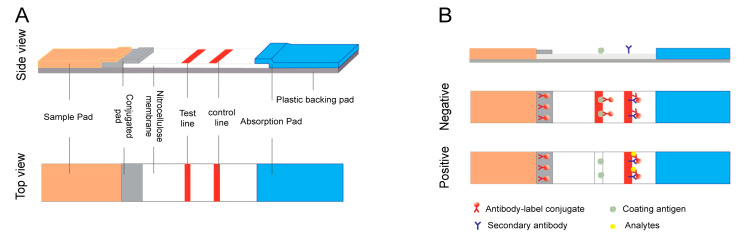
Schematic illustration of the lateral flow immunoassay (LFA) test strip. (**A**) Typical composition of an LFA test strip, and (**B**) principles of competitive LFA testing.

A typical LFA device contains four sections (sample pad, conjugate pad, nitrocellulose membrane, and absorbance pad) which are laminated in an orderly manner onto a sheet of plastic backing (Figure 2A). Various formats are possible depending on the type of target analyte [21], but sandwich and competitive assays are the two standard formats most frequently used.

### 2.1. Sandwich Format

Sandwich assays are typically used for high molecular weight molecules or targets with multiple antigenic sites such as proteins and bacteria. Generally, this system uses a specific antibody pair, as the test line is coated with the unlabeled antibody, and the detected antibody is used to bind the signal label. When the sample is added to the test strip, the specific antibody reacts with the labeled antibody–target complex to form a clear test line through sample migration. The response detected in the test zone is directly proportional to the number of targets in the sample. The mixture then passes through the capture zone, where both the unbound and bound analytes bind to the capture antibody [22,23].

### 2.2. Competitive Format

Competitive assays are employed most often when testing low molecular weight analytes or when presenting a single antigenic determinant. For this format, the analyte–protein complex is immobilized on the test line, competing with the target in the sample to conjugate the labeled antibody. The response detected on the test line is negatively proportional to the amount of the target in the sample. Another specific antibody bound to the control line allows for the capture of excess antibody complex. Therefore, a band color appears in the control line regardless of the presence of the target analyte, confirming that the test was run correctly (Figure 2B) [22,23].

The test strip is a one-step procedure and the liquid sample to be analyzed is placed on the sample pad. All membrane pads are usually made with nitrocellulose. The reagent membrane comprises the immobilized specific antibodies and the labeled antibodies. With the addition of the sample, the reacting molecules are solubilized and combined with the expected metrics in the sample. Then, capillary action directs the fluid mixture to the reaction membrane [21].

## 3. Applications for Detecting β-Adrenergic Agonist Residues

β-adrenergic agonists are a group of synthetic phenethanolamine compounds, and according to their different aromatic ring structures they can be separated into three categories: aniline, phenol, and resorcinol [24]. LFA technology has been adequately developed to detect β-adrenergic agonists. As they are low molecular mass compounds, competitive assays should be used, the principles of which were described in Section 2 of this review (2. LFA formats and principles). If the analyte is not present in the sample, then the labeled antibody will be caught by the bovine serum albumin (BSA)-analyte and immobilized on the membrane to form a clear test line with negative results; if the analyte is present in the sample exceeding the lower detectable concentration, it competes with the BSA-analyte immobilized on the test line and binds to the finite amount of labeled antibody, leading to an invisible test line with positive results. Published reports on LFA applications in this field are summarized in Table 1.

Various reagents can help visualize antigen–antibody interactions, but gold nanoparticles (AuNPs) are most frequently used in test strips. When many AuNPs aggregate, their color changes notably, and their application in colorimetric immunoassays is based on this principle [43]. AuNP-based LFAs have been used to detect β-adrenergic agonists. Zhang et al. [28] used AuNP-based LFA devices to detect clenbuterol in swine urine, as it was demonstrated that the limit of detection (LOD) for the device was 0.1 ng/mL when using the scanner and 1.0 ng/mL by eye, with the test completed within 10 min. In addition, Lai et al. [26] developed a lateral flow assay to detect CLE in swine livers. The results obtained when using this assay were compared to those of a commercial ELISA kit (the Ridascreen Clenbuterol Fast Kit [R1701, R-Biopharm, Darmstadt, Germany]), which is used to detect clenbuterol. Khamta et al. [31] developed a lateral flow assay to detect salbutamol. They used a salbutamol-BSA conjugate as the test line, goat antirabbit IgG as the control line, and an anti-SALB-colloidal gold conjugate (SALB-CGC) as the detector. The sensitivity to salbutamol in PBS is approximately 80 ng/mL. An AuNPs-based LFA was proposed by Liu et al. [32] to detect ractopamine (RAC) residues in swine urine. The LOD for the LFA strip, when using an optical density scanner, was 0.1 ± 0.013 ng/mL and the cut-off level was 1.0 ng/mL when observed with the naked eye. These data show that the lateral flow strip assay is a reliable screening method.

Zvereva et al. [33] proposed an indirect labeling method that could be applied with an LFA to detect SAL residues in food. The visual LOD was 4.0 ng/g for meat products and 3.0 ng/g for milk samples, and there was a high level of cross-reactivity with terbutaline (50%) and orciprenaline (10.5%). Xu et al. [44] prepared monoclonal antibodies against 12 β-adrenergic agonists (salbutamol, clenbuterol, brombuterol, clenpenterol, mabuterol, carbuterol, cimbuterol, mapenterol, pirbuterol, terbutaline, cimaterol, and clenproperol), which were applied to AuNPs-based LFA devices. The visual LOD values were 1, 1, 2.5, 2.5, 2.5, 2.5, 2.5, 5, 10, 10, 5, 10, and 10 ng/mL, respectively, and detection was completed within 5–10 min.

Quantitative detection is usually performed using the optical density (OD) of the signal detected in the test line [28,32]. However, the ODs in the test line also change with pH value, temperature, and reaction time. Therefore, test results determined solely by the strength of the test line will be inaccurate [30]. Li et al. [30] established a quantitative lateral flow immunoassay for the detection of CLE in porcine urine. The ODs of the test and control zones on the test strip were measured to compensate for the inherent factors of the strip and the influence of the sample matrix, with the LOD being as low as 220 pg/mL. Similarly, Ren et al. [34] used this method for the quantitative detection of ractopamine in swine urine.

LFA devices that use other colored NPs have been proposed for signal labeling. For example, an LFA device which used selenium nanoparticles (SeNPs) as labels was developed by Wang et al. [35] for the detection of CLE. The cut-off value for the visual detection of CLE in swine urine was 3 ng/mL. Wang et al. [36] also established a lateral flow immunoassay for the detection of RAC and SAL in swine urine using SeNPs as probes and simultaneously detected both on one test strip. The visual detection limits for RAC and SAL were 1 and 3 ng/mL, respectively. Yu et al. [37] used red and blue silica nanoparticles (SiNPs) as visible labels for the detection of CLE (red) and RAC (blue) for the first time, and their LODs were 3 and 2 ng/mL, respectively. Zhu et al. [38] proposed the use of a core-shell purple SiNP-based LFA for CLE screening via the naked eye, and the cut-off values were 3, 6, and 5 ng/mL in phosphate buffered saline, urine, and pork, respectively.

The sensitivity of these methods equals that of AuNPs-based methods. However, in recent years, researchers have developed more advanced LFA devices to detect β-adrenergic agonists on-site.

## 4. Novel Labels for Use in LFAs to Detect β-Adrenergic Agonist Residues

To further enhance the detection sensitivity of LFAs, a variety of labeling materials, such as luminescent NPs, enzymes and nanozymes, magnetic NPs (MNPs), and quantum dots, have been used to detect β-adrenergic agonist residues; Table 2 summarizes the pros and cons of different labels applied in LFAs and the published reports on the new labels are summarized in Table 3.

### 4.1. Quantum Dots

During recent decades, the development of quantum dots (QDs) has aroused considerable interest in labeling materials, which have been used as biosensors due to their advanced characteristics, including size-adjustable light emissions, excellent fluorescence intensity, powerful resistance against photo-bleaching, narrow excitation spectrum, and wide emission spectrum [71]. For example, Luo et al. [45] established a QD-based LFA device for the rapid detection of CLE. However, the LOD for CLE is 30 ng/mL and the sensitivity of the strip was lower than the previously described LFA using AuNPs. The reason for this may be that the excess antibody is not completely removed, so more CLE is required to saturate the QD-labeled antibody, which directly reduces the sensitivity of detection.

### 4.2. Luminescent Nanoparticles

**Figure 3:**
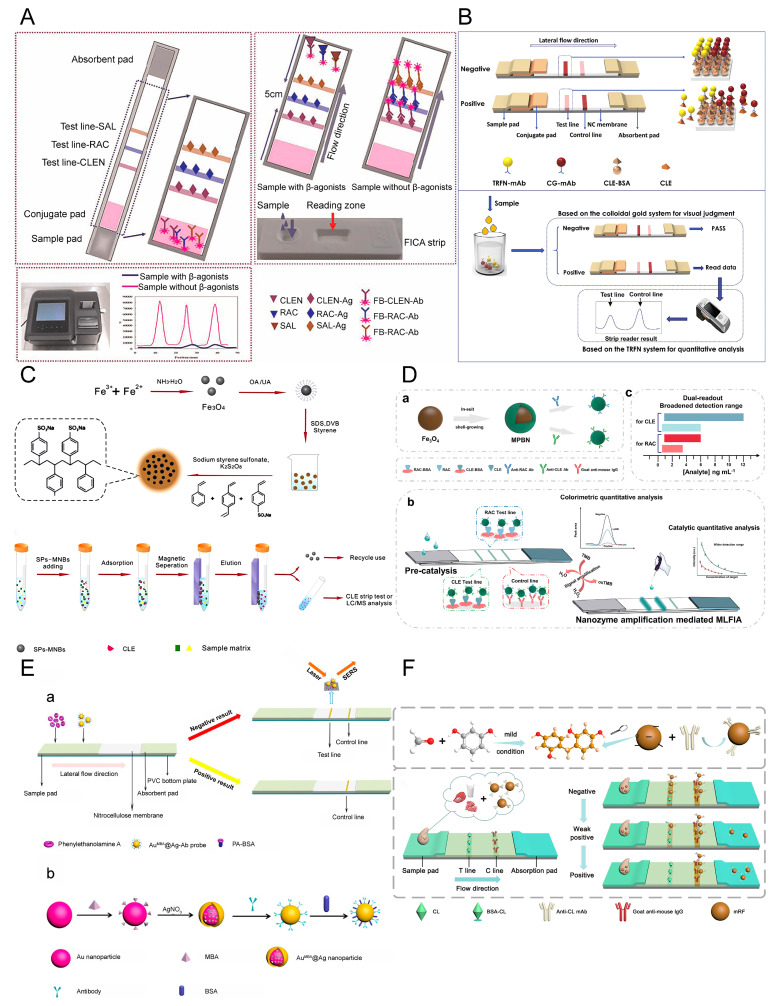
Novel labels for LFAs. Schematic illustrations showing: (**A**) a multicomponent fluorescent LFA device, reprinted with permission from [46], Copyright 2014, Elsevier; (**B**) an LFA using AuNPs and TRFN as labels for the detection of CLE, reprinted with permission from [37], Copyright 2019, Elsevier; (**C**) spMNB preparation and CLE adsorption from pork muscle samples, reprinted with permission from [55], Copyright 2014, Elsevier; ((**D**)-**a**) the synthesis process for magnetic Prussian blue nanozymes (MPBN), ((**D**)-**b**) principles of the LFA strip when using MPBN as the signal system, ((**D**)-**c**) detection range comparison in dual-read mode, reprinted with permission from [50], Copyright 2020, Elsevier; ((**E**)-**a**) SERS-based LFA for PA detection and ((**E**)-**b**) preparation of the Au^MBA^ @ Ag-Ab probe, reprinted with permission from [51], Copyright 2014, American Chemical Society; (**F**) schematic illustration showing the constitution of an mRF-mAb probe and mRF-based LFA device, reprinted with permission from [67], Copyright 2021, Elsevier.

Fluorescent microspheres (FMs) are special polystyrene microspheres with fluorescent substances within them [72]. Deng et al. [53] described an LFA device that uses fluorescein isothiocyanate polystyrene-fluorescent microspheres as probes to quantitatively detect CLE in swine urine, in which they also introduced a sample pre-incubation strategy; each 100 μL swine urine sample was added to the plate containing FMs-mAb and incubated at room temperature for 5 min, and then dropped the mixture into the sample well of the test paper. The LOD with this method was 0.01 pg/L, and it took 20 min to complete, with a recovery of 85.0–107.5%. Wang et al. [46] reported a fluorescent multicomponent LFA device for the simultaneous quantitative detection of CLE, RAC, and SAL (Figure 3A), with the results acquired in less than 10 min without sample pretreatment. The LOD for CLE, RAC, and SAL was 0.10, 0.10, and 0.09 ng/mL, respectively, and the recovery rate was 70.0–100.5%, with a relative standard deviation of <15%. LFAs that used FMs had a higher sensitivity for the detection of CLE compared to AuNPs. There was no cross-reaction between CLE, RAC, and SAL. It is worth noting that although cimaterol, mabuterol, and terbutaline showed a 10% cross-reaction on the SAL test line, this slight cross-reaction had very limited effects on the specificity of the test strip.

### 4.3. Lanthanide

Most fluorescent signals are only present for a short time, and ‘time resolved’ refers to the time delay required to separate the target fluorescence signal from the interfering background fluorescence. Lanthanide chelates are a group of luminescent materials, and a time-resolved fluoroimmunoassay based on lanthanide labeling offers many advantages over traditional fluorescence assays, such as larger Stokes shifts, longer fluorescence lifetime, sharper emission spectra, and lower background signal interference [73]. This means that the sensitivity of lanthanide-labeled LFAs could be significantly improved [46]. Eu (III) [Eu^3+^] is one of the most commonly used lanthanide labels in time-resolved fluorescent LFAs.

Song et al. [7] coated porous silica nanoparticles with Eu(III)-BHHCT to form steadily fluorescent nanosilica with satisfying luminescent characteristics, establishing an LFA device to detect CLE. The visual LOD for qualitative detection of this LFA device was 0.1 ng/mL, while the LOD for quantitative detection was reduced to 0.037 ng/mL with the use of a fluorescent biosensor. The detection time did not exceed 8 min, which is suitable for on-site rapid detection. Chen et al. [37] integrated LFAs for the qualitative and quantitative detection of CLE using AuNPs and time-resolved fluorescent nanobead (TRFN) as markers (Figure 3B). With qualitative testing, negative samples could be quickly excluded with the naked eye. The positive samples were further quantitatively detected in the same strip. The AuNP probe optimizes the range of linearity of the TRFN probe. Hu et al. [54] systematically compared the quantitative detection of RAC in swine urine using TRFN, FMs, QDs, and AuNP-based lateral flow assays. The TRFN-LFA had the highest sensitivity of 7.2 pg/mL and showed a wide linear range from 5 to 2500 pg/mL. Each FM-LFA, QD-LFA, and CG-LFA test strip used 0.02, 0.054, and 0.15 μg anti-RAC poly antibody (pAb), respectively, whereas only 0.005 µg of pAb was used in the TRFN-LFA test strip. Furthermore, TRFN-LFA required the least RAC-BSA antigens and performed the shortest assay test time compared to the other three test strips. The results exhibited those obtained by TRFN-LFA, followed by those obtained by LC-MS/MS, and commercial ELISA kits.

### 4.4. Up-Conversion Nanoparticles

Up-conversion NPs (UCNPs) can convert lower energy sources into higher energy luminescent emissions. Because the analysis has no background luminescence, time-resolved detection is not required, which simplifies the process [74,75]. Furthermore, background absorption can be minimized as there is anti-Stokes luminescence; no photo-degradation of biomolecules occurs due to excitation in the infrared region [71]. Therefore, when compared with fluorescent organic dyes and QDs, UCNPs have several advantages, such as higher photo-stability, a longer fluorescence lifespan, lower cost, and lower cytotoxicity [43]. Wang et al. [47] adopted a UCNP-based LFA device for the sensitive detection of ultra-trace CLE in animal urine, tissues, and feed samples. The visual LOD for UCNP-LFA for CLE was 0.1 ng/mL, yet could be as low as 0.01 ng/mL using the test strip reader. The results showed that the sensitivity of the UCNPs was notably higher than the AuNPs [25] and FMs [46], with high specificity.

### 4.5. Magnetic Nanoparticles

MNPs are a promising nanomaterial with unique advantages when used as labels in LFAs. MNPs can not only separate targets from the matrix under the action of a magnetic field to reduce matrix effects and increase the concentration of the target but can also provide optical and magnetic signals [76]. Iron oxide nanoparticles are the least toxic MNPs and thus the most common in the biomedical field. Wu et al. [55] synthesized novel sulfonated polystyrene magnetic nanobeads (spMNBs), which pre-concentrated the β-adrenergic agonists commonly found in pork, and used them combined with an AuNP-based LFA for quantitative detection (Figure 3C). This method simplified the sample preparation and shortened the extraction time from 90 min to 30 min. Huang et al. [48] established a sensitive LFA based on fluorescent magnetic nanobeads (FMNBs) for the detection of CLE in swine urine. The method integrated immunomagnetic separation and fluorescent LFA to eliminate matrix effects and improve sensitivity. FMNBs have a Fe_3_O_4_@ SiO_2_@ QDs structure. FMNB-based probes served both as carriers for sample enrichment and as fluorescent labels in the LFA, where the two modular steps were seamlessly linked. Consequently, the device had only one probe and no elution steps that could lead to a loss of the target. The linear range was 0.25–5.00 ng/mL in swine urine, and the LOD was 0.22 ng/mL, which is four times higher in sensitivity than the AuNP-based LFA. The results demonstrated that the established LFA device had resistance to the matrix of swine urine and had good accuracy in multiple urine samples, which could be applied to the on-site detection of actual samples.

### 4.6. Enzymes and Nanozymes

Several biomolecules can also serve as labels in LFAs. Enzymes are the most used biosignal molecules as they have strong catalytic properties, which enable them to catalyze the redox reactions that convert substrates into colored products, further amplifying the signal and enhancing sensitivity. Furthermore, this allows labels to be detected directly by the naked eye or by a highly sensitive reader. In biological assays such as ELISA, Western blot, and LFA, the most used enzymes are horseradish peroxidase (HRP) and alkaline phosphatase. Gao et al. [49] proposed an LFA that used HRP-labeled antibodies as probes for the rapid detection of both RAC and SAL. The signal decreased with the increase of RAC and SAL concentrations due to the competition format used. Under optimal conditions, the linear ranges of RAC and SAL were 0.5–40.0 ng/mL and 0.1–50.0 ng/mL, and the LOD values were 0.20 ng/mL and 0.04 ng/mL, respectively, with high specificity. Wang et al. [56] established an LFA capable of the simultaneous quantitative detection of RAC and CLE. In this study, RAC and CLE antibodies were labeled with HRP and ALP as signal probes; after competitive immune actions were completed on the test line, the test line was cut off and placed in a reaction cell containing substrates which comprised luminol and P-iodophenol (PIP). Finally, 20 mL of newly prepared co-reactant containing hydrogen peroxide and ALP substrate was added. The chemiluminescence signals of RAC and CLE were detected at 3 and 300 s after the addition of the co-reactant. The LOD for RAC and CLE were 0.17 ng/mL and 0.067 ng/mL, respectively, and the entire detection process was completed within 20 min.

Some NPs have darker original colors and similar peroxidase activity to HRP. Compared with natural enzymes, nanozymes have the advantages of being highly stable, enabling visual observation and convenience. Therefore, nanozymes can be used as ideal signal labels for LFAs [77,78]. Prussian blue nanoparticles (PBNPs) are environmentally friendly and readily bind to antibodies without additional activators [79]. Liu et al. [50] developed a novel multiplex LFA device for the simultaneous detection of RAC and CLE using MPBNs as the signal system (Figure 3D). The bifunctional MPBN label comprised an Fe_3_O_4_ core and a PBNPs shell. Magnetic particles served as substrates to enhance the visual color of the PBNPs and simplify the separation step during antibody labeling. After adding tetramethylbenzidine (TMB) solution to the test line, the color of the test lines turned blue due to the peroxidase mimicking the catalytic activity of MPBN, and the color intensity was negatively proportional to the concentration of RAC and CLE. The colorimetric signal generated by the primary color of the tag and the catalytic signal generated by the oxidation of the catalytic color substrate TMB, led to higher accuracy and wider detection ranges, and there was almost no cross-reaction between the two analyte targets. The calculated limits of detection (cLOD) for RAC and CLE were 0.12 ng/mL and 0.20 ng/mL, respectively, and the detection ranges were 1–6 ng/mL and 1–12 ng/mL, respectively, after TMB-H_2_O_2_ was added.

### 4.7. Surface-Enhanced Raman Scattering-Active Nanomaterials

Surface-enhanced Raman scattering (SERS) is an ultra-sensitive trace-analysis technique. When particles are adsorbed onto the surface of a precious metal, the Raman signal can be amplified using SERS technology [80]. AuNPs and AgNPs are considered ideal labels for quantitative LFAs due to their unique, localized surface plasmon resonance characteristics and, consequently, they are widely used as SERS-active nanomaterials [81,82]. However, SERS enhancement of AuNPs is relatively weak. Reporter molecules absorbed on the surface of AuNPs are susceptible to environmental interference [83], leading to inaccuracies in the detected signals, which is a limitation when conducting trace analysis.

Core-shell nanostructures can generate robust and steady SERS enhancement and have been used as labels in quantitative LFAs [84]. Au@ Ag core-shell nanoparticles exhibit high SERS activity, which is chiefly due to the electronic ligand effect and localized electric field enhancement in core-shell nanoparticles [85]. Phenylethanolamine A (PA) is a novel β-agonist, and Li et al. [51] synthesized Au@ Ag-Ab by immobilizing anti-PA antibodies onto the surface of Au/Ag core-shell nanoparticles that contain a Raman reporter (Figure 3E). This nanomaterial was used as a probe for LFA detection of PA in urine with an LOD of 0.32 pg/mL. The group subsequently reported similar LFAs based on the Au@ Ag core-shell nanotags for the detection of CLE, SAL, and brombuterol, with an LOD as low as 0.24, 3.0, and 0.5 pg/mL, respectively, with detection completed within 15 min [59,60,61]. Su et al. [60] reported a novel bimetallic core-shell Au/Au nanostar and used it as a SERS tag in a colorimetric/SERS dual-model LFA for the detection of CLE residues. They encapsulated the Raman reporter molecule 5,5′-dithiobis (2-nitrobenzoic acid) (DTNB) between the core (AuNP) and the shell (Au nanostar), which could eliminate environmental interference and improve stability. The design of the Au star shell created many sharp tips and increased surface roughness, which improved the sensitivity of the SERS analysis, and the limit of quantitation detection was 0.05 ng/mL. Some interferences were selected to verify the specificity; only CLE could abolish the color and SERS intensity on the T line. The results demonstrated that the SERS-based LFA is a rapid, simple, ultra-sensitive and highly specific assay device.

### 4.8. Other Novel Labels

Dye-doped nanoparticles contain many dye molecules inserted in a silica matrix and possess a stronger fluorescent signal than organic fluorophores. They are ideal for trace biological analysis without extra reactant agentia or signal amplification steps. Xu et al. [62] applied Ru(phen)32+ doped silica nanoparticles as fluorescent probes to LFA test strips for the rapid quantitative screening of five common β-agonist (SAL, CLE, simbuterol, terbutaline, and brombuterol) residues in swine urine. The LOD of SAL was 0.43 ng/mL, and the sensitivity significantly improved. The device can be used for broad spectrum screening of β-agonist residues in swine urine.

Graphene nanoparticles have been applied in various fields due to their excellent properties and economic benefits. Huang et al. [63] used graphene nanometers instead of traditional AuNPs as signal probes to label BSA-CLE in an LFA device to detect CLE in foods of animal origin. As the graphene nanoparticles had a strong adsorption capacity for BSA, only one simple mixing step was required, which was economical and efficient. The visual LOD was 0.1 ng/mL, which improved sensitivity when compared with traditional markers. In addition, Liu et al. [86] labeled AuNPs, nanogold-polyaniline-nanogold microspheres (GPGs), and colloidal carbon with anti-salbutamol (SAL) antibodies, respectively. The performance of the three nanoparticle labels in SAL detection was systematically and comprehensively compared. The experimental results showed that the stability of GPG and colloidal carbon was better than the AuNPs. Due to the low cost of colloidal carbon, the LFA based on colloidal carbon was an inexpensive device for the rapid detection of SAL on-site and could be mass-produced.

Prussian blue nanoparticles (PBNPs) typically range in size from a few nanometers to several hundred nanometers, which is much larger than traditional immunolabels, resulting in a more pronounced color band in the test line, increasing the sensitivity. Zhao et al. [64] developed a simple LFA using PBNPs as markers, which was successfully applied for CLE detection in pork, pig kidney, and bacon samples. The detection results demonstrated that the sensitivity of the device was 5-fold higher than AuNP-based LFAs, and it exhibited an excellent linear relationship for CLE between 0.5 and 5.0 ng/mL.

Coomassie brilliant blue (CBB), which can bind to protein with high affinity, is a widely used protein staining method used for quantitative analysis. The probe based on CBB has the advantages of requiring only simple preparation, being low cost, and having a higher competitive efficiency for antigens compared with traditional LFA. Zhang et al. [65] proposed an LFA that used CBB-staining antibodies as both recognition signals and chromogenic probes to detect CLE in food, with a two-fold sensitivity higher than AuNP-based LFAs.

Ultramarine blue nanoparticles are a good substitute for AuNPs, due to their low cost, higher yield in comparison with AuNPs and other nanoparticles, and their characteristics of having a bright color, good stability, and not easily fading. Liu et al. [66] conjugated ultramarine blue nanoparticles with antibodies as probes and signaling molecules in LFA for the on-site detection of RAC. Ultramarine nanoblue accumulation produces a very bright blue signal, showing good sensitivity and specificity.

Resorcinol formaldehyde resin polymer (RF) is an inexpensive cross-linked polymer with the advantages of a large surface area and good biocompatibility, and it has been used in many fields. Its variety of chemical groups means it can be combined with antibodies without additional modification and activation, and due to the large specific surface area of RF, it can exhibit bright color signals on the NC membrane. Wang et al. [67] proposed a method to synthesize mild resorcinol formaldehyde resin polymer (mRF) at room temperature (30 °C), which simplifies the synthesis process and avoids the release of formaldehyde due to high temperatures during the reaction process (Figure 3F). mRF can be directly combined with antibodies by electrostatic adsorption. When mRF was applied as the signal label in an LFA to detect CLE, the LOD was 1 ng/mL, the sensitivity was four times higher than traditional AuNPs-based LFA, and the recovery rate was 96.7–117.2%.

**Figure 4:**
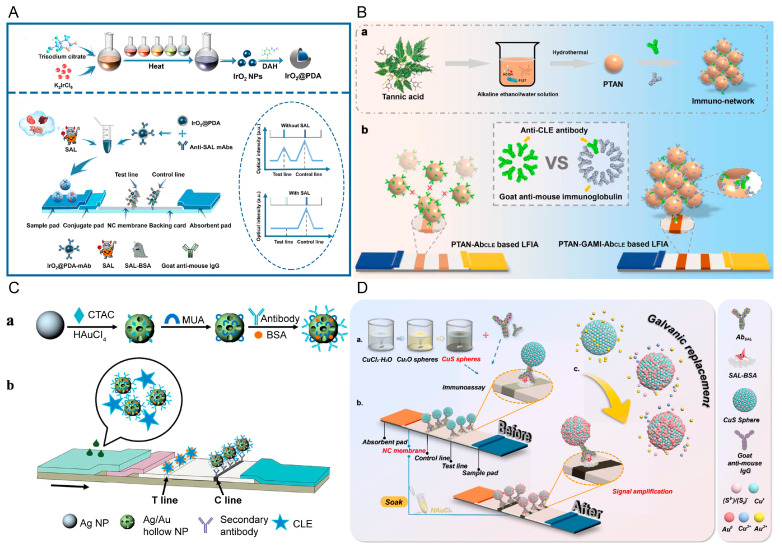
Novel labels used to improve the sensitivity of LFAs. Schematic illustrations showing: (**A**) synthesis of IrO_2_@ PDA and IrO_2_@ PDA-based LFAs, reprinted with permission from [68], Copyright 2021, American Chemical Society; ((**B**)-a) preparation of a bioresource-derived PTAN-based immuno-network and ((**B**)-b) comparison of an indirect probe-based immune network with a traditional direct label format, reprinted with permission from [69], Copyright 2022, Elsevier; ((**C**)-a) preparation and surface modifications of hollow Au-Ag NPs and ((**C**)-b) a schematic illustration of hollow Au-Ag NP-labeled LFAs, reproduced from [61]; (**D**) Schematic illustration of CuS-NS-based signal amplification, reprinted with permission from [70], Copyright 2022, Elsevier.

The synthesis of iridium oxide nanoparticles (IrO_2_ NPs) is simple, and they have a high level of chemical stability. They are thus a promising marker with a high surface-area-to-volume ratio, a high level of catalytic activity, and are corrosion resistant. However, IrO_2_ NPs are very small, and this may lead to the partial loss of monoclonal antibodies (mAbs) during the labeling process. Polydopamine (PDA) has an abundance of functional groups on the surface and has a high affinity for a variety of metals, which can be easily attached to its surface. Therefore, PDA can modify metal oxide nanoparticles to form a core-shell morphology, and the NPs covered with a PDA coating not only improve the affinity to the substrate but also enhance the biocompatibility and the color intensity of the nanoparticles that are suitable for biological testing [87]. Zhao et al. [68] developed an LFA with dopamine-modified iridium oxide nanoparticles (IrO_2_@ PDA NPs) as signal labels to detect SAL in pork, pork liver, and beef (Figure 4A). IrO_2_@ PDA exhibited a higher mAb affinity and stronger optical signal. The LOD for SAL was 0.002 ng/mL, and its sensitivity was 24-fold and 180-fold higher than the LFA based on IrO_2_ NPs and AuNPs, respectively, with high specificity.

Li et al. [69] used poly tannic acid nanospheres (PTANs) as a novel marker and proposed an immune network-based indirect labeling system for LFAs supported by bioresource-derived tannic acid (TA) for CLE detection (Figure 4B). The PTANs were synthesized using a formaldehyde-assisted cross-linking strategy. Due to its strong protein enrichment ability, PTAN can directly conjugate goat anti-mouse immunoglobulin (GAMI) on its surface and then combine with anti-CLE antibodies to form an immune network. Because the existence of GAMI improves the utilization rate of the anti-CLE antibody, the sensitivity was also improved. Compared with PTAN-based LFA and AUNPs-based LFA, the sensitivity was increased by at least 5-fold and 10-fold, respectively.

Compared with AuNPs, the surfaces of gold nanoflowers (AuNFs) are rougher and have larger surface-to-volume ratios, higher signal intensities under natural light, and a stronger ability to bind to antibodies. Lai et al. [52] developed AuNF-labeled LFAs integrated with smartphones to detect CLE in swine urine. Smartphones could efficiently collect, read out, and distinguish subtle changes in the signal from the test leads; as they are simpler, cheaper, and more user-friendly than expensive readers, which are relatively complex, they may be a promising alternative to optical readers. The sensitivity for this device was 4.9-fold higher than the AuNP-LFA in PBS with a LOD of 12.5 pg/mL, and in swine urine, it was 59.0 pg/mL, with a linear range of 0.1–5.0 ng/mL. The average recovery for AuNF-labeled LFAs was 92.7–112.0%, and the coefficients of variation (CV) were 1.8–7.4%.

Wang et al. [61] manufactured bimetallic, hollow, gold-silver nanoparticles using the galvanic replacement (GR) reaction, and they were found to have larger reaction areas, lower cytotoxicity, and excellent optical properties compared with AuNPs (Figure 4C). A series of parameters for Au-Ag NPs were optimized to amplify the visual detection signal and quantitative data. The visual detection limit was better than traditional AuNP test strips. In this work, the visual sensitivity of the LFA for the detection of CLE using the hollow Au-Ag NP label could be as low as 2 ng/mL. In recent years, the use of copper (Cu) deposition-assisted signal amplification with LFAs has progressed, and is now used for the on-site ultra-sensitive analysis of biomolecules [88]. However, due to the use of the necessary reducing agent, there are inherent defects in the application of LFAs, such as strong background interference to the NC film, self-nucleation during storage, and low levels of reproducibility. Shu et al. [70] used GR to inspire the in-situ growth of AuNPs on CuS nanospheres (CuS-NS), which was first used in an LFA device to detect SAL in pork and beef (Figure 4D). GR relies on the redox potential differences to drive metal ions to form sediment on other inorganic surfaces, without reducing agents, which effectively avoids the above-mentioned shortcomings. When compared with excess CuS-NS, the number of antibodies used was reduced two-fold, and the sensitivity was increased by five times while reducing the cost.

## 5. Strategies to Increase the Performance of LFA Systems

### 5.1. Novel RAC-BSA Carrier Conjugation

In LFA devices based on the competition format, a small molecule antigen must be bound to the protein carrier, which is immobilized on the NC membrane as a solid-phase antigen, with BSA usually used as the carrier protein. An RAC-BSA conjugate has reportedly been developed based on the Mannich reaction with a 9:1 molar ratio of RAC-BSA [89]. Compared with other methods, this coupling method has many advantages, such as stability, a simple one-step process, and it requires fewer reagents. A novel RAC-BSA-based LFA test strip was used to detect RAC in animal feed. LOD values calculated by the reader were as low as 0.1 ng/g and did not cross-react with related compounds.

### 5.2. Different Sample Addition Methods

**Figure 5:**
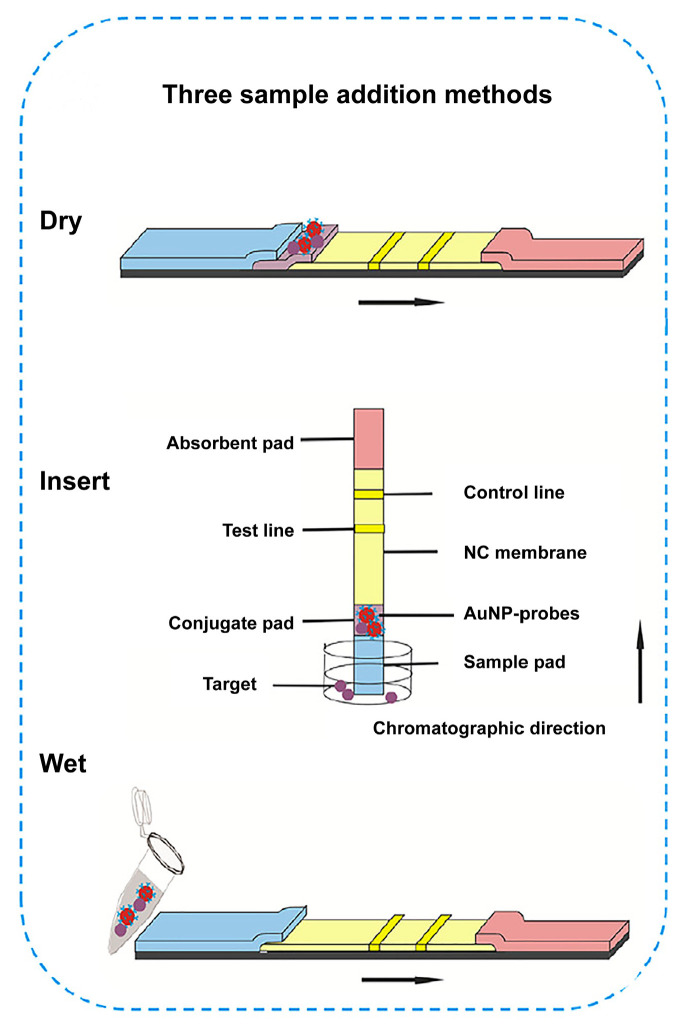
Comparison of three sample addition methods (dry, wet, and insert), reprinted with permission from [90], Copyright 2020, Elsevier.

There are three methods used to add samples to LFA devices, namely dry, wet, and insert models. The dry method is achieved by directly adding a certain amount of sample solution dropwise to the sample well of the LFA detection kit. The wet method is performed by adding a pre-incubated mixture of probe and sample dropwise to the sample well of the LFA strip. While the insert method is accomplished by dipping the sample pad of the LFA device directly into the solution to be tested (Figure 5). The dry and insert methods are the most common sample addition methods for the LFA test strips, whereas the wet method is more common in scientific research. Li et al. [90] systematically investigated the effects of the three sample addition methods on the detection performance of AuNP-based LFA devices, including reading time, linear range, LOD, and CV. For the competitive LFA strips with CLE as an analyte, the difference between the three methods in the ideal PBS solution was negligible; whereas in pork samples, the wet method showed the worst performance, but the CV value of the wet method was lower than the dry and insert method. This study can be used as a reference, providing guidance on how to select a suitable sample addition method.

### 5.3. Multiplex Detection of β-Adrenergic Agonists

Besides improving the sensitivity of an LFA, it is also important to improve detection efficiency and reduce costs. It is thus necessary to develop LFAs that can detect multiple β-adrenergic agonists simultaneously. To achieve this, researchers developed an LFA labeled with AuNPs to simultaneously detect CLE and RAC in swine urine [25]. The LOD for the CLE and RAC was 0.1 ng/mL, respectively, when using the OD scanner. Wu et al. [29] developed a two-directional LFS strip for the simultaneous detection of CLE, SAL, and RAC. The two-directional LFA technique could effectively avoid cross-reactions, and the detection limit of the three analytes was 0.5 ng/mL.

Novel markers and those with increased sensitivity have also been used in multiplex detection LFAs. Peng et al. [91] synthesized Au nanoclusters (AuNCs) using 6-aza-2-thiothyamine (ATT) and L-arginine (Arg), and obtained high green luminescence efficiency and ultra-small nanoparticles (Arg/ATT/AuNC). A multiplex lateral flow immunoassay (AuNCs-MLFA) device based on highly luminescent green AuNCs with two detection lines was established for the simultaneous detection of CLE and RAC residues in swine urine. The guanidine group of Arg could combine with ATT; therefore, the carboxyl group of the Arg/ATT/AuNCs could easily conjugate with biomolecules and enhance the fluorescence intensity. The Arg/ATT/AuNCs showed strong green luminescence under a UV lamp, and the visual detection limits of CLE and RAC were both 0.25 ng/mL. When a portable fluorescence reader was used, the ratio of the fluorescence intensity for the test and control lines was used as a quantitative signal, and the LOD values were 0.003 and 0.023 ng/mL, respectively. Compared with AuNP-based LFAs, the developed AuNC-MLFA method exhibited excellent performance [91]. Similarly, researchers have also used fluorescent nanoparticles [46], enzymes and nanozyme [49,50,56], selenium nanoparticles [36], and silica nanoparticles [37,62] as labels for LFAs to achieve the multiplex detection of β-adrenergic agonists with satisfactory results.

### 5.4. Fluorescence Quenching

**Figure 6:**
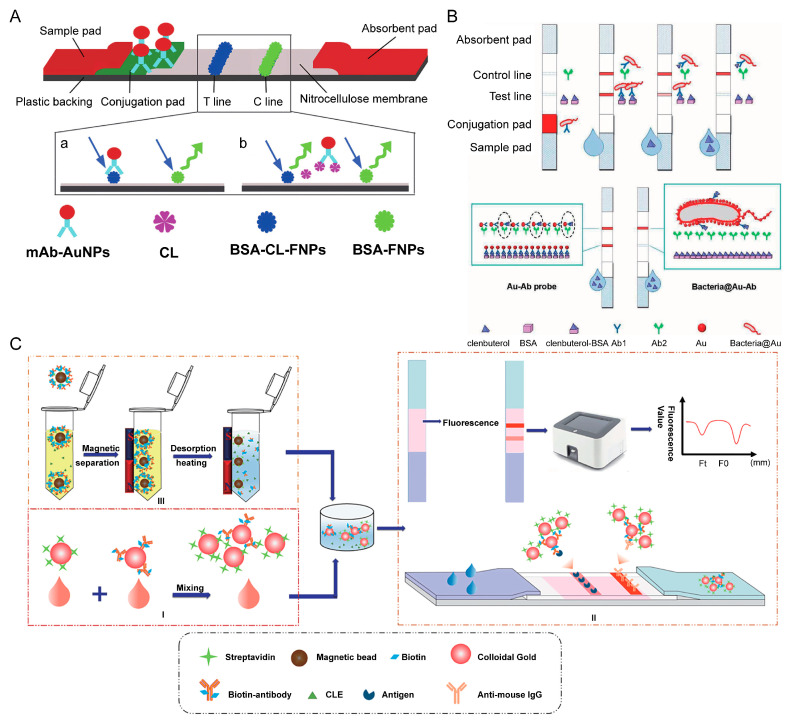
Strategies for signaling amplification. ((**A**)-a) Fluorescent LFA under “turn-on” mode and (b) AuNP-LFA under “turn-off” mode, reprinted with permission from [92], Copyright 2014, Elsevier; (**B**) schematic illustration of LFA with bacteria@ Au composite as a signal amplifier, reprinted with permission from [95], Copyright 2018, Elsevier; ((**C**)-I) scheme for improving the sensitivity of an LFA using AuNPs labeled with biotinylated antibody and (II) streptavidin, background fluorescence block-ing, and (III) immunomagnetic separation, reprinted with permission from [96], Copyright 2019, Springer Nature.

Because β-adrenergic agonists are small molecules, the traditional LFA test strips only use a single type of nanoparticle to detect them and this is based on a competitive format, where the signal intensity is negatively proportional to the concentration of the target, which is called the “turn-off” mode. Tang et al. [92] established a more sensitive “turn-on” LFA based on the fluorescence-quenching effect of AuNPs. Under an excitation light, the fluorescent labels on the test line emit fluorescence; the signal intensity is proportional to the analyte concentration, and the fluorescence disappears in the presence of the quenching agent. The principle of the “turn-on” mode is shown in Figure 6A. AuNPs are not only used as labels but also as the fluorescence quencher. LFAs were used to detect CLE in swine urine. The sensitivity of the traditional AuNP-based “turnoff” LFA was 5 ng/mL when using the naked eye under natural light, and the sensitivity of the “turn-on” LFA under excitation light was 0.08 ng/mL. The group also developed a fluorescent quenching LFA test strip for RAC detection with LOD values as low as 0.16 ng/mL [93]. Zhang et al. [94] prepared a “turn-on”-patterned LFA based on the same principle. They used fluorescein isothiocyanate FMs as fluorescent labels and AuNPs as fluorescence quenching agents. When the coated antigen was in conjunction with the antibody, AuNPs caused quenching of the fluorescence signal on the test line. The LFA test strip was used to detect clorprenaline (CLP), and the LOD value was 0.12 ng/mL, with the results obtained within 15 min.

### 5.5. Bacteria@ Au Composites

When fewer antibodies were applied to the competition-based LFA device, the sensitivity to the analyte increased, and the signal intensity was weaker. Therefore, reducing antibody volumes while generating sufficient signals is a challenge when developing LFAs. Huang et al. [95] proposed a probe using inactivated bacteria as the carrier for the AuNPs (Figure 6B). Many AuNPs can be enriched on the surface of the bacteria, but as they only bind to a few antibodies, the sensitivity is improved. When compared with other labels, bacteria can be obtained using simple cultures without complex chemical synthesis, which is an ideal signal amplifier. They applied bacteria@ Au-Ab composites to LFAs to detect CLE and the visual LOD was 0.1 ng/mL; the sensitivity was 20-fold higher than conventional LFA devices.

### 5.6. Biotin-Streptavidin System

Biotin-streptavidin is a signal amplification system commonly used in immunoassays. The specific binding of biotin and streptavidin effectively reduces steric hindrance to achieve signal amplification, and due to its small size, it does not impede the binding site of the antibody while binding to it. Researchers have developed an LFA device to detect trace CLE in swine urine, using biotinylated antibody and streptavidin-AuNP conjugates instead of Ab-AuNP conjugates; the combination of fluorescence quenching technology and immunomagnetic separation not only improved the sensitivity of the LFAs, but also reduced the matrix effect of the sample [96] (Figure 6C). The linear range of the test strip for CLE was 0.06–0.59 ng/mL, and the LOD was 0.25 ng/mL with the naked eye, with a limit of quantitative detection of 0.03 ng/mL; the sensitivity was 60 times higher than the traditional LFA.

## 6. Microfluidic Paper-Based Analytical Devices

Microfluidic technology originated in the early 1990s [97]. Although the device was small, use of the microfluidic apparatus based on polydimethylsiloxane was primarily confined to the laboratory. This was because the microfluidic that the devices used also required many external pumps, valves, and other instruments, which reduced on-site detection capabilities and increased the cost. Paper is an inexpensive and ubiquitous material, and microfluidic paper-based analytical devices (μPADs) are the latest generation of lab-on-a-chip devices. Using paper as a substrate for microfluidic devices can effectively reduce the cost and is also advantageous due to its wide availability, simple fabrication, easy disposability, and having no requirement for external pumps. The performance difference between μPAD and LFA is conspicuous (Table 4). Unlike LFAs, μPADs use chemical printing and/or cutting to create flow channels. Therefore, traces of samples can be used for high-throughput analysis. Consequently, this method has attracted great interest from researchers and has become a promising analytical platform.

Whitesides’ laboratory released the first microfluidic paper-based analytical device (µPAD) in 2007 [19], which heralded the beginning of the µPAD field of research. Currently, µPADs are extensively used in clinical diagnosis, environmental monitoring, and food analysis [98,99,100,101]. The Whitesides group has used photo-lithography to accurately create hydrophobic barriers around hydrophilic channels in paper using photo-resistance. The µPAD was used to detect glucose and protein in artificial samples, and their concentrations were proportional to the color signal intensity [19]. Although photo-lithography is an effective micropatterning technique, the complex manufacturing procedures, expensive photo-lithography equipment, and photo-resist reagents limit its large-scale production and application. There has been rapid development in this field and many fabrication strategies have been introduced to replace photo-lithography and develop designs for two-dimensional (2D) and three-dimensional (3D) μPADs, including wax printing and dipping [102,103], pen plotting [104], inkjet printing [105], PDMS plotting [106], plasma treatments [107], laser treatments [108], stamping [109], flexographic printing [110], and cutting [111]. These methods are used to pattern hydrophilic membranes on paper to create hydrophobic barriers so samples and reagents can flow through isolated areas. Previous reviews have discussed the pros and cons of the fabrication techniques for µPADs [112]. The choice of fabrication method depends on many factors such as production cost, material substrate, and fabrication speed [113]. For the selection of paper substrates, several physical properties of the paper, which include thickness, porosity, and wicking speed, affect the sample transfer rate within the microfluidic device. Whatman filters are made from pure cellulose and are popular for their uniform thickness and wicking characteristics [114,115].

**Figure 7:**
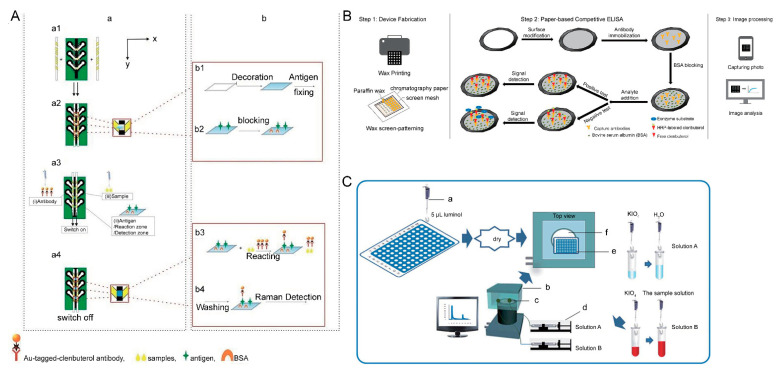
Schematic illustrations showing: ((**A**)-a) the configuration and work-flow of a slide-engaged manual switch-on-chip multilayer paper chip and ((**A**)-b) the reaction process of the CLE detection zone, reprinted with permission from [119], Copyright 2015, John Wiley and Sons; (**B**) paper-based competitive ELISA for the detection of CLE in foods, reprinted with permission from [116], Copyright 2018, Elsevier; and (**C**) the μPAD fabrication process using the wax-printing method, ((**C**)-a) pipettor; ((**C**)-b) CL reader; ((**C**)-c) injection well; ((**C**)-d) syringe pump; ((**C**)-e) PAD and ((**C**)-f) photomultiplier. reprinted with permission from [117], Copyright 2014, The Royal Society of Chemistry.

μPADs have been used for the detection of β-adrenergic agonists [116,117,118,119]. Ma et al. [116] developed a paper-based microfluidic competitive ELISA for the detection of CLE in water and milk by combining it with a μPAD (Figure 7B), and the LOD was 0.2 ppb. The detection zones on the paper were defined using wax printing or wax screen-patterning techniques. Compared with the traditional 96-well plate ELISA, the analysis time was reduced, as was the number of reagents, which is an improvement on the traditional ELISA.

For the detection of β-adrenergic agonists, urine or blood samples from animals are usually used. However, these samples are difficult to collect and store, and β-adrenergic agonists in urine and blood have a short half-life and rapid metabolism, which can easily lead to false negative test results. As hair is readily available and β-adrenergic agonists are present in hair for longer periods than in urine [120], recent studies have used animal hair to detect β-adrenergic agonists. Chen et al. [117] combined µPAD with chemiluminescence (CL) to develop a µPAD device for the rapid and quantitative detection of β-adrenergic agonists in swine hair. The β-adrenergic agonists decreased the CL produced by the reaction of luminol and potassium periodate solution on the μPAD, and reduction was proportional to the concentration of the β-adrenergic agonists (Figure 7C). The device was designed using a 96-well plate format and could detect 48 samples simultaneously. Subsequently, the group also proposed a novel chemiluminescence-based μPAD device [118], which was fabricated using a wax-printing method and used K_3_ [Fe (CN) _6_] and luminol as the chemiluminescence reagents. The method had a wide linear range, low detection limit, and required fewer samples and reagents, with a recovery of 78–95%. Zheng et al. [119] proposed a 3D μPAD for the detection of CLE in swine hair. The 3D structure of the sensor improved the repeatability and sensitivity of the microfluidic devices. In this study, the antigen was immobilized on the patterned paper, and the 3D structure was obtained by stacking or folding patterned paper along the vertical axis, and a pair of paper slides were inserted into the slot on the chip as a switch to control how the samples flowed. The controllable multi-step interaction between the swine hair extract analyte and SERS probe-modified CLE antibody and antigens could be realized by maneuvering the flow of the two fluidic samples, which was achieved for SERS immunoassay detection on the chip (Figure 7A). In addition, researchers also found that the Whatman No. 1 filter paper was the best substrate for the SERS immunoassay detection of CLE. The LOD of this 3D μPAD for CLE was 0.1 pg/mL, and the detection performance was satisfactory.

## 7. Conclusions and Future Perspectives

β-adrenergic agonists (i.e., “lean meat powder”) pose a huge threat to human health, and their use has consequently been banned repeatedly in animal husbandry. However, because of their continued illegal use, timely on-site detection has become important to enable accurate monitoring. Paper is inexpensive and ubiquitous, making it an ideal substrate for sensors. In this review, we have described recent advances in paper-based devices for the rapid on-site detection of “lean meat powder” and discussed fabrication methods and detection modalities. LFA test strips are the most popular paper-based analytical devices due to their advantages, which include being portable, fast, convenient, and low cost. The labels used in LFA systems are traditionally and predominantly AuNPs. However, researchers have developed various new labeling and signal amplification strategies for use in LFAs to comprehensively improve system performance.

Despite the rapid development of LFA technologies over the past decade, there are still problems that need to be addressed. In the LFA apparatus, samples flow laterally on the NC membrane via capillary force, and the total reaction time depends on the flow speed taking 10–20 min for detection. To obtain sufficient flow speed, the pore size of the paper is limited to several micrometers, which limits the capture and analysis sensitivity of the biomolecules. Furthermore, if the proportion of antigens and antibodies is inappropriate, this can lead to a false negative result, called the hook effect, and when the LFA test strip has multiple test lines, it is easily interfered with by a cross-reaction due to its geometry [121]. The VFA device offers an improvement on the performance of the LFA, and the most important difference between the two methods is their vertical and lateral flow of fluids. Reports on the design and development of VFAs indicate that they have been applied for clinical purposes, environmental monitoring, and food safety. The basic principle of the VFA is the same as for the LFA. The labeled antibodies are immobilized on the conjugate pad, and detection is accomplished through antigen–antibody specific binding [20]. However, VFAs have several advantages over LFAs, the most significant of which is that the fluid flow rate is faster, and the detection time is reduced. The VFA device uses a sensing membrane with isolated immunoreaction spots for multiple detections. As the channels are spatially isolated, there can be no cross-reaction, and this has powerful multiplexing capabilities. Due to its superior performance, the VFA device is worthy of further investigation and could be used for on-site testing [20]. Future work should consider adding reagent reservoirs to VFAs to automate the loading of reagents to meet commercial requirements and modify the geometric shape of VFAs to develop a POCT device with a multiplexed-detection format. Although LFA devices have been commercialized for the detection of β-adrenergic agonist residues, VFA devices can fully compete in the market. The focus when developing new devices for the detection of “lean meat powder”, should be on VFAs and their commercialization for high-throughput detection on-site as soon as possible.

Furthermore, regardless of whether LFAs or VFAs are used to detect β-adrenergic agonists, the biological molecules employed for the identification of the targets in both systems are antibodies. Antibodies are proteins and thus are susceptible to temperature, pH, and other factors, which will lead to denaturation, impacting their effects. Moreover, the cost of antibodies is relatively high. Aptamers are single-stranded DNA or RNA oligonucleotide fragments, usually obtained via the systematic evolution of ligands by exponential enrichment (SELEX). While the process of aptamer selection may be expensive, once the aptamers are screened, they can be synthesized with high reproducibility and purity, resulting in the cost of aptamers being appreciably lower than antibodies [122]. Aptamers undergo conformational changes when they bind to targets and can bind to targets with high specificity and affinity. Compared with antibodies, they have advantages such as stability, low cost, and a short screening period, and have been used to detect small molecules [123]. Aptamers offer a promising alternative to antibodies and are expected to help to generate probes for β-adrenergic agonist detection in the field.

Since the Whitesides group published the first μPAD article in 2007, the field has flourished. μPADs have a high sensitivity, with fast and high-throughput detection, and can be used without using external pumps, creating a reliable analysis platform that could replace LFAs. The application of μPADs for the on-site detection of “lean meat powder” is still in the early stages of development. Compared with LFAs, μPADs have higher sensitivity and stronger high-throughput detection abilities. For commercialization, it will be necessary to propose new paper-based materials to obtain more biocompatible substrates, as well as improved micropatterning techniques to enhance overall performance while reducing costs. Additionally, understanding the principles of capillary flow in μPADs will help to build better analysis patterns.

To achieve the commercialization and large-scale production of paper-based analytical devices for the on-site detection of “lean meat powder”, the transformation of scientific and technological achievements should be accelerated. Firstly, the fabrication and application process should be simplified so untrained users can easily employ the devices. In addition, appropriate sample pretreatment methods, novel signal labeling and signal amplification strategies need to be developed to achieve higher sensitivity. The detection format for paper-based devices needs to be refined to make them more portable, faster, and reliable. In addition, smartphones which facilitate a vital role in peoples’ lives, could also provide a new development direction for on-site analysis, and the combination of smartphones with analysis methods has broad developmental prospects. Finally, companies should comply with the relevant regulations, maintaining the quality of products while reducing costs.

In summary, due to the growth in food demand, the new generation of “lean meat powder” detection devices for on-site analysis must have the characteristics of being low cost, fast, simple, portable, and accurate. A paper-based analysis is evidently a promising approach that could revolutionize this process.

## Figures and Tables

**Table 1:** Summary of the analytical performance of traditional paper-based LFAs for the detection of β-adrenergic agonists.

Analyte	Label	Assay Format	Sample	Assay Time	LOD	Reference
CLE ^1^ and RAC ^3^	AuNPs ^2^	Competitive LFA	Swine urine	5 min	0.1 ± 0.01 ng/mL	[25]
CLE	AuNPs	Competitive LFA	Swine livers	10 min	NA	[26]
CLE	AuNPs	Competitive LFA	Swine urine	10 min	3 ng/mL	[27]
CLE	AuNPs	Competitive LFA	Swine urine	10 min	0.1 ng/mL	[28]
CLE, RAC, SAL ^4^	AuNPs	Competitive LFA	Swine urine	10 min	0.5 ng/mL	[29]
CLE	AuNPs	Competitive LFA	Swine urine	10 min	220 pg/mL	[30]
SAL	AuNPs	Competitive LFA	Swine urine	10 min	80 ng/mL	[31]
RAC	AuNPs	Competitive LFA	Swine urine	5 min	0.1 ng/mL	[32]
SAL	AuNPs	Competitive LFA	Meat and milk	10 min	meat: 4.0 ng/g milk: 3.0 ng/g	[33]
RAC	AuNPs	Competitive LFA	Swine urine	45 min	0.13 ng/mL	[34]
CLE	SeNPs ^5^	Competitive LFA	Swine urine	/	3 ng/mL	[35]
RAC and SAL	SeNPs	Competitive LFA	Swine urine	5 min	RAC: 1 ng/mL SAL: 3 ng/mL	[36]
CLE and RAC	SiNPs ^6^	Competitive LFA	/	10 min	CLE: 3 ng/mL RAC: 2 ng/mL	[37]
CLE	SiNPs	Competitive LFA	PBS, urine, and pork	10 min	PBS: 3 ng/mL urine: 6 ng/mL pork: 5 ng/mL	[38]
Zilpaterol	AuNPs	Competitive LFA	Feed	10 min	20 ng/g	[39]
PA ^7^	AuNPs	Competitive LFA	Swine urine	10 min	0.188 ng/mL	[40]
Clorprenaline	AuNPs	Competitive LFA	Swine urine	3–5 min	0.104 ng/mL	[41]
Clorprenaline	AuNPs	Competitive LFA	Swine urine	9 min	0.15 ng/mL	[42]

^1^ CLE: clenbuterol. ^2^ AuNPs: gold nanoparticles. ^3^ RAC: ractopamine. ^4^ SAL: salbutamol. ^5^ SeNPs: selenium nanoparticles. ^6^ SiNPs: silica nanoparticles. ^7^ PA: phenylethanolamine.

**Table 2:** Summary of the pros and cons of different labels applied in LFAs.

Labels	Advantages	Disadvantages	Reference
AuNPs	Ease of fabrication, good biocompatibility, direct observation, stability	Low sensitivity	[25]
QD	Strong resistance against photo-bleaching, narrow excitation spectrum, wide emission spectrum	Need reader for quantitation; toxic	[45]
FM	Strong fluorescence, low cost	Easy to photo-bleach; aggregation-caused quenching	[46]
Lanthanide	Large Stokes shift, low background signal interference, long fluorescence lifetime	Need reader for quantitation	[7]
UCNPs	High photo-stability, long fluorescence lifespan, low cost, low cytotoxicity	Need reader for quantitation	[47]
MNPs	Reduced matrix effects, increased the concentration of the target	Low signal intensity	[48]
Enzymes	Able to catalyze the redox reactions	Easy to decomposition, high cost	[49]
Nanozymes	Ease of fabrication, low cost, stability	Need reader for quantitation	[50]
SERS	Good biocompatibility, stability, simple preparation, high sensitivity	Need reader for quantitation	[51]

**Table 3:** Summary details for LFAs using novel labels for β-adrenergic agonist residues.

Analyte	Label	Sample	Linear Range	Assay Time	Detection Limit		Reference
					Qualitative	Quantitative	
CLE	AuNFs ^1^	Swine urine and PBS	0.1–5.0 ng/mL	10 min	/	12.5 pg/mL	[52]
CLE	QD ^2^	PBS	/	/	/	30 ng/mL	[45]
CLE	FM ^3^	Swine urine	/	20 min	/	0.01 pg/L	[53]
CLE, RAC, SAL	FM	Swine urine	0.0–4.0 ng/mL	10 min	/	CLE: 0.10 ng/mL RAC: 0.10 ng/mL SAL: 0.09 ng/mL	[46]
CLE	Fluorescent nanosilica	Swine urine	/	8 min	0.1 ng/mL	0.037 ng/mL	[7]
CLE	AuNPs and fluorescent nanobead	Pork	0.1–2.7 ng/mL	5–20 min	0.5 ng/mL	0.04 ng/mL	[37]
RAC	TRFN ^4^	Swine urine	5–2500 pg/mL	10 min	/	7.2 pg/mL	[54]
CLE	UCNPs ^5^	Swine urine	/	10 min	0.1 ng/mL	0.01 ng/mL	[47]
CLE	spMNBs ^6^ and AuNPs	Pork muscle	0.05–1.20 ng/mL	40 min	0.10	0.24 ng/g	[55]
CLE	FMNBs ^7^	Swine urine	0.25–5.00 ng/mL	10 min	/	0.22 ng/mL	[48]
RAC, SAL	HRP ^8^	Swine urine	RAC: 0.5–40.0 ng/mL SAL: 0.1–50.0 ng/mL	20 min	/	RAC: 0.20 ng/mL SAL: 0.040 ng/mL	[49]
RAC, SAL	HRP, ALP ^9^	Swine urine	/	20 min	/	RAC: 0.17 ng/mL CLE: 0.067 ng/mL	[56]
RAC, CLE	MPBN ^10^	Pork and mutton	RAC: 1–6 ng/mL CLE: 1–12 ng/mL	10 min	/	RAC: 0.12 ng/mL CLE: 0.20 ng/mL	[50]
PA	SERS ^11^	Swine urine	/	15 min	/	0.32 pg/mL	[51]
CLE	SERS	Swine urine	0–10 ng/mL	15 min	/	0.24 pg/mL	[57]
SAL	SERS	Swine feed, meat, and urine	10^−4^–100 ng/mL	15 min	/	3.0 pg/mL	[58]
brombuterol	SERS	Pork and swine urine	/	15 min	/	0.5 pg/mL	[59]
CLE	SERS	Meat	0–1 ng/mL	8 min	5 ng/mL	0.05 ng/mL	[60]
CLE	Au-Ag NPs	PBS	/	15 min	/	2 ng/mL	[61]
SAL	Ru(phen)32+ doped silica NPs	Swine urine	0.6–5.0 ng/mL	15 min	/	0.43 ng/mL	[62]
CLE	Graphene NPs	Meat	0.1–2.0 ng/mL	10 min	0.1 ng/mL	0.05 ng/mL	[63]
CLE	PBNPs ^12^	Meat	0.5–5.0 ng/mL	15 min	1.0 ng/mL	/	[64]
CLE	CBB ^13^	Animal products	2–10 ng/mL	10 min	/	2 ng/mL	[65]
RAC	Ultramarine blue NPs	Feed, pork	/	10 min	feed: 2 ng/mL pork: 1 ng/mL	/	[66]
CLE	mRF ^14^	Animal products	0–2 ng/mL	15 min	1 ng/mL	/	[67]
SAL	IrO_2_@ PDA NPs ^15^	Food samples	0.02–3.00 ng/mL	15 min	/	0.002 ng/mL	[68]
CLE	PTAN ^16^	Beef and pork liver	0.0–0.9 ng/mL	15 min	0.6 ng/mL	0.13 ng/mL	[69]
SAL	CuS@ Au ^17^	Beef and pork	5.0–12.0 ng/mL	10 min	/	4.0 μg/kg	[70]

^1^ AuNFs: gold nanoflowers. ^2^ QD: quantum dot. ^3^ FM: fluorescent microsphere. ^4^ TRFN: time-resolved fluorescent nanobead. ^5^ UCNPs: up-conversion NPs. ^6^ spMNBs: sulfonated polystyrene magnetic nanobeads. ^7^ FMNBs: fluorescent magnetic nanobeads. ^8^ HRP: horseradish peroxidase. ^9^ ALP: alkaline phosphatase. ^10^ MPBNs: magnetic Prussian blue nanozymes. ^11^ SERS: surface-enhanced Raman scattering. ^12^ PBNPs: Prussian blue nanoparticles. ^13^ CBB: Coomassie brilliant blue. ^14^ mRF: mild resorcinol formaldehyde resin polymer. ^15^ IrO_2_@ PDA NPs: dopamine-modified iridium oxide nanoparticles. ^16^ PTAN: poly tannic acid nanospheres. ^17^ CuS@ Au: CuS nanospheres and Au nanoparticles.

**Table 4:** Compared performance between LFAs and μPADs.

Features	LFAs	μPADs
Sample flow	Capillary force	Capillary force
Washing steps	No	Yes
Multiplexing capability	Moderate	High
Hook effect	Yes	No
Easy-of-use	Easy	Moderate
Sensitivity	Moderate	High
Sample volume requirement	∼µL	∼µL

## Data Availability

Not applicable.
